# Differential Effects of Tra2ß Isoforms on HIV-1 RNA Processing and Expression

**DOI:** 10.1371/journal.pone.0125315

**Published:** 2015-05-13

**Authors:** Craig Platt, Maria Calimano, Josip Nemet, Jodi Bubenik, Alan Cochrane

**Affiliations:** Dept. of Molecular Genetics, University of Toronto, Toronto, Ontario, Canada; Istituto Superiore di Sanita, ITALY

## Abstract

Balanced processing of HIV-1 RNA is critical to virus replication and is regulated by host factors. In this report, we demonstrate that overexpression of either Tra2α or Tra2β results in a marked reduction in HIV-1 Gag/ Env expression, an effect associated with changes in HIV-1 RNA accumulation, altered viral splice site usage, and a block to export of HIV-1 genomic RNA. A natural isoform of Tra2β (Tra2ß3), lacking the N-terminal RS domain, also suppressed HIV-1 expression but had different effects on viral RNA processing. The functional differences between the Tra2β isoforms were also observed in the context of another RNA substrate indicating that these factors have distinct functions within the cell. Finally, we demonstrate that Tra2ß depletion results in a selective reduction in HIV-1 Env expression as well as an increase in multiply spliced viral RNA. Together, the findings indicate that Tra2α/β can play important roles in regulating HIV-1 RNA metabolism and expression.

## Introduction

Regulation of alternative splicing is thought to play a critical role in the function of a cell. Predictions are that >90% of the mRNAs in the human genome undergo alternative splicing (reviewed in [1, 2]). Thus, alternative splicing allows the sequence complexity of the genome to be dramatically increased from the ~25,000 genes currently predicted. This point is particularly true for HIV-1, the integrated provirus generating a single 9 kb transcript that is processed by alternative splicing to generate ~40 mRNAs to produce the proteins essential for new virion formation [3, 4]. The viral RNAs generated are grouped into three classes, unspliced (US), singly spliced (SS) and multiply spliced (MS). Expression of proteins encoded by US and SS RNAs is dependent upon export mediated by the HIV-1 factor, Rev [5, 6]. Disruption of HIV-1 RNA processing severely inhibits HIV-1 gene expression and replication [3, 4]. One component in the regulation of RNA alternative splicing is represented by the host cell SR proteins. SR proteins are characterized by the presence of one or two amino-terminal RNA recognition motifs (RRM) and a C-terminal domain rich in alternating arginine and serine residues (RS domains) (reviewed in [7]). SR proteins are also involved in constitutive splicing, mRNA export, stability and translation [7, 8]. The SR protein family can be extended to include other RS domain containing proteins that are termed SR-related proteins (reviewed in [9]). Unlike SR proteins, SR-related proteins are unable to support constitutive splicing in S100 extracts.

Analysis of cis-acting sequences regulating HIV-1 RNA splicing have determined that use of a specific splice site is determined in part by the relative activity of adjacent exon splicing enhancers (ESEs) and exon spicing silencers (ESSs) that act in an antagonistic fashion by binding SR or hnRNP proteins, respectively [3, 4]. Two of the ESEs within HIV-1, GAR and ESE3, contain purine-rich sequences [3, 10] that are known binding sites for the human SR-related Tra2α and Tra2β proteins [11–17], homologues of the *Drosophila melanogaster* splicing regulatory factor Tra2 [18–22]. Both proteins have a similar organization; N-terminal and C-terminal regions rich in arginine and serine separated by an RNA recognition motif (RRM). Their sequences are 75% identical, with the differences occurring mainly in the first 49 amino acids of the protein and in the position of a polyglycine region in the C-terminal RS domain [23]. Isoforms of both proteins have been observed. In the case of Tra2α, a variant comprised of only the central RRM domain has been detected upon screening of ESTs, while alternative splicing of Tra2β RNA yields five different mRNAs but only two proteins, the full length (Tra2β1, NCBI RefSeq NM_004593.2) and a variant lacking the N-terminal RS domain (Tra2β3, NCBI RefSeq NM_001243879.1) (the other three spliced variants generating reading frames with premature termination codons that prevent expression) [19, 24–26]. Both Tra2α and Tra2β can regulate alternative splicing [27] by binding purine-rich splicing enhancer elements. In some instances, Tra2β interacts with a member of the SR or hnRNP family to alter the splicing of the target RNA [12, 14, 15, 17, 26, 28].

Given their potential to interact with known ESEs within HIV-1, we were interested in exploring whether Tra2α/β played a significant role in regulating HIV-1 RNA processing and expression. To address this question, we used both overexpression and depletion approaches. Mutagenesis was also used to examine the contribution of the various Tra2α and Tra2β protein domains to protein localization and regulation of splicing of HIV-1 and another model RNA. In this report, we demonstrate that the N- and C-terminal RS domains of Tra2α and Tra2β differ in their capacity to regulate RNA processing in the context of HIV-1. Overexpression of Tra2α/β and a variant lacking the N-terminal RS (Tra2α/βΔN, equivalent to Tra2β3) suppressed expression of HIV-1 Gag and Env through the nuclear sequestration of the viral RNAs that was reversed upon Rev overexpression. However, the WT and ΔN constructs had distinct effects on HIV-1 RNA splicing. Functional differences among the structural variants were also observed in the context of another model RNA (doublesex). In contrast, deletion of the C-terminal RS domain or point mutations in the RRM of Tra2α/β had little or no effect on HIV-1 gene expression. Subsequent depletion studies demonstrated a role for Tra2β in the regulation of HIV-1 gene expression, reduction in its expression resulting in a selective decrease in viral Env protein and an increase in MS RNA with only limited effects on Gag protein and the accumulation of viral unspliced (US) or singly spliced (MS) RNAs.

## Materials and Methods

### Plasmids

The plasmid HIV Hxb2 R-/RI- was provided by E. Cohen and contains mutations inserting two stop codons between the protease and reverse transcriptase regions of the virus. The plasmid pNL4-3 GagzipGFP was generated by replacement of the NC, PR and RT coding regions of pNL4-3 with a leucine zipper sequence fused to GFP using an In-Fusion (Clontech) cloning strategy. Blencowe (University of Toronto) generously provided the plasmids pLexA-Tra2α, pLexA-Tra2β, pADML dxsΔ434, pADML dsx (GAA)_6_, and pADML dsx MS2. CMVmyc3xterm was previously described [29]. Bl-Tra2α was generated using PCR primers 5’CCCAAGTCCA GCGACAGCGG CGAGCAGA 3’ and 5’ GCTCTAGAAG CACCAAACTA CACAAA 3’. The resulting amplicon was cloned into the *Eco*RV site of BLSK to create Bl-Tra2α. CMVmycTra2α was constructed by digesting Bl-Tra2α with *Hind*III / *Xba*I and cloning the resulting fragment into the respective sites in CMVmyc3xterm. CMVmycTra2αΔN was generated by digesting CMVmycTra2α with *Xmn*I /*Xba*I and ligating the fragment into CMVmyc3x term cut with *Eco*RV/*Xba*I. CMVmycTra2αΔNΔC was created by digesting CMVmycTra2α with *Xmn*I /*Eco*RI and ligating the fragment into CMVmyc3xterm cut with *Eco*RV /*Eco*RI. CMVmycTra2αΔC was produced by digesting CMVmycTra2α with *Hind*III/*Eco*RI and ligating into the *Hind*III/*Eco*RI sties of CMVmyc3xterm. CMVmycTra2αffdd was created by QuickChange Mutagenesis (Stratagene) using the following primers: 5’ CGATCTCGAG GAGACGCTGA CGTGTATTTT GAG 3’ and 5’CTCAAAATAC ACGTCAGCGT CTCCTCGAGA TCG 3’ and CMVmycTra2α as template DNA according to manufacturer’s protocol. CMVmycTra2β was generated by digesting pLexA-Tra2ß with *Xho*I followed by blunting with Vent polymerase and subsequent digestion with *Eco*RI. This fragment was then ligated into the *Eco*RI/*Sma*I sites of CMVmyc3xterm. CMVmycTra2βΔN was generated by PCR using the following primers 5’GGAATTCTTT AATAGCGACG AGGTGAG 3’ and 5’CCCAAGCTTA TGTCTACTCG CAGGCGTCAT 3’. The amplicon was cut with *Hind*III/*Eco*RI and cloned into the *Hind*III/*Eco*RI sites of CMVmyc3xterm. CMVmycTra2βΔNΔC was created by PCR using the following primer pair 5’CCCAAGCTTA TGTCTACTCG CAGGCGTCAT 3’ and 5’CGGAATTCGC AGCTCTCCAT CCTCCTCC 3’. The resulting amplicon was cut with *Hind*III/*Eco*RI and ligated into the *Hind*III/*Eco*RI sites of CMVmyc3xterm. CMVmycTra2βΔC was made by PCR with the following primers 5’CCCAAGCTTA TGAGCGACAG CGGC 3’ and 5’GCGAATTCTA CGCCCATCAA GCTC 3’. The amplicon was cut with *Hind*III/*Eco*RI and ligated into the respective sites of CMVmyc3xterm. To generate GFP fusions of each factor, Sac1/BamH1 fragments of the CMVmyc Tra2β, Tra2βΔN, Tra2βΔC, or Tra2βffdd were cloned into the Sac1/BamH1 sites of peGFP-C1. The SV-1 env construct was provided by H. Schaal [30] and the SV-1 envΔES variant was generated using the QuickChange Mutagenesis PCR protocol (Stratagene) using the primers 5’-ACA GGC CCG AAG GAA TAG GAT CCT TGG CAC TTA TCT-3’ and 5’-AGA TAA GTG CCA AGG ATC CTA TTC CTT CGG TGT-3’ to delete ESE3 and ESS3.

### Western blots

3x10^5^ HEK293T cells were transfected with plasmids using a calcium phosphate protocol [31]. Forty-eight hours posttransfection, cells were washed and harvested in PBS. The cells were pelleted by centrifugation at 5000 rpm for 5 minutes and the pellet was resuspended in 30 μl of SDS-PAGE sample buffer (0.125M Tris-HCl, pH 6.8, 4% SDS, 20% glycerol, 0.2% bromophenol blue). The samples were boiled for 4 minutes and fractionated on 12.5% SDS-PAGE gels. Proteins were transferred onto PVDF membranes, the resulting blots washed with 0.05% Tween in PBS and blocked with 5% milk in PBS for 30–60 minutes. Proteins were detected using anti-HIV-1 p24^CA^ hybridoma supernatants [32], anti-HIV-1 Env hydriboma antibody [33], anti-tubulin (Sigma), anti-Tra2β (Abcam, ab31353), or anti-myc (9E10) antibodies followed by HRP-conjugated anti-mouse antibody (Jackson Laboratories) and the signal developed using Western Lightning Plus (Perkin Elmer Life Sciences). To assess the relative level of transfected and endogenous proteins, blots were also probed with rabbit anti-Tra2β antibody generously provided by Stephan Stamm.

### Immunofluorescence

HeLa and HEK 293 were transfected with 2.0 μg of the plasmids using a calcium phosphate protocol as above. Forty-eight hours posttransfection, cells were rinsed with PBS and fixed in 4% paraformaldehyde, 1X PBS for 30 minutes at room temperature. The cells were washed twice with 10mM glycine in PBS and permeabilized with 1% Triton X-100 in PBS for 5 minutes. After 2 washes, cells were blocked in 3% BSA in 1x PBS for 1 hour at room temperature. The coverslips were then inverted over the primary antibodies and incubated for 1 hour in a humidified chamber. The cells were washed again and incubated with fluorescently labeled secondary antibodies for 1 hour. Following washing, the coverslips were incubated with 0.05 μg/ml of DAPI in PBS for 5 minutes to allow detection of the nuclei. Samples were mounted onto slides using a solution of 2-phenylenediamine in 100mM Tris pH 8.0, 90% glycerol. Images were obtained using OpenLab software on a Leica DMR microscope and captured with a Hamamatsu CCD camera. Anti-myc antibody was obtained from Invitrogen. Anti-SRm300 was provided by B. Blencowe. Staining for the HA epitope used supernatant from the 12CA5 hybridoma.

### RNA Analysis

HEK 293T cells were seeded at a density of 1.5 x 10^6^ and transfected with 1 μg of pAdML dsx plasmids or pHxb2 R-/RI- plasmid and 4 μg of the indicated Tra2 vectors. Forty-eight hours post-transfection, total RNA was isolated according to Chomczynski and Sacchi [34]. Northern blotting to detect alteration on HIV-1 RNA levels was performed as previously outlined [35]. For RT-PCR, cDNA was generated by incubation of 3 μg of total RNA with T_20_VN and M-MLV reverse transcriptase as outlined by the manufacturer (Invitrogen). Analysis of HIV-1 RNA splicing was performed as previously described [36, 37]. qRT-PCR analysis of HIV-1 RNA levels was performed as previously outlined [37]. For the analysis of dsx RNA, PCR was performed using the primers dsxF (5’ GCT GAT GCC ACT CAT GTA TG 3’) and dsxR (5’TGA CGG GAG TAC TCA TTC AC3’). Cycling conditions for dsx amplification were 94 C for 30 s, 60 C for 30 s, and 72 C for 30 s for 30 cycles.). Amplicons were separated on native, 5% polyacrylamide gels. Quantitation of unspliced/spliced dsx RNA ratios was performed using ImageQuant software following exposure of gels to phosphor screens and scanning with a Molecular Dynamics PhosphorImager.

To assess the impact of the Tra2β variants on HIV-1 US RNA subcellular distribution, 293 cells were transfected with peGFP C1-fusions along with pHxb2 R-/RI-, processed, and probed with Stellaris^TM^ FISH probes consisted of a mixture of 48 Quasar 570-labelled 20-mer oligonucleotides spanning the HIV-1 Gag coding region and were used as detailed by the manufacturer (Biosearch Technologies). Images were captured using a Leica DMR epifluorescence microscope at 630X magnification.

### shRNA depletion of endogenous Tra2β

To examine the effect of Tra2ß depletion on HIV-1 gene expression, the HeLa rtTA HIVΔmls cell line [37] was transduced with lentiviral virus generated by transfection of 293T cells with the packaging vector pAX2, VSV-G and either pLKO vectors expressing a control shRNA or one against Tra2β (target sequence 5’-TGCCGATGTGTCTATTGTATA-3’). After 24 h, virus was removed and transduced cells selected by treatment with 1–2 μg/ml puromycin for 2 days. Following selection, HIV-1 virus expression was induced by addition of 2 μg/ml doxycycline and cells harvested for protein and RNA analysis after 24 h.

#### Statistical analysis

Data was analyzed by Microsoft Excel and expressed as means ± standard deviation. Differences relative to control were compared by two-tailed Student’s *t*-test. Values determined to be significant (p<0.05) are marked by an asterisk.

## Results

### Effect of domain deletions on Tra2α and Tra2β expression and subcellular localization

**Fig 1:**
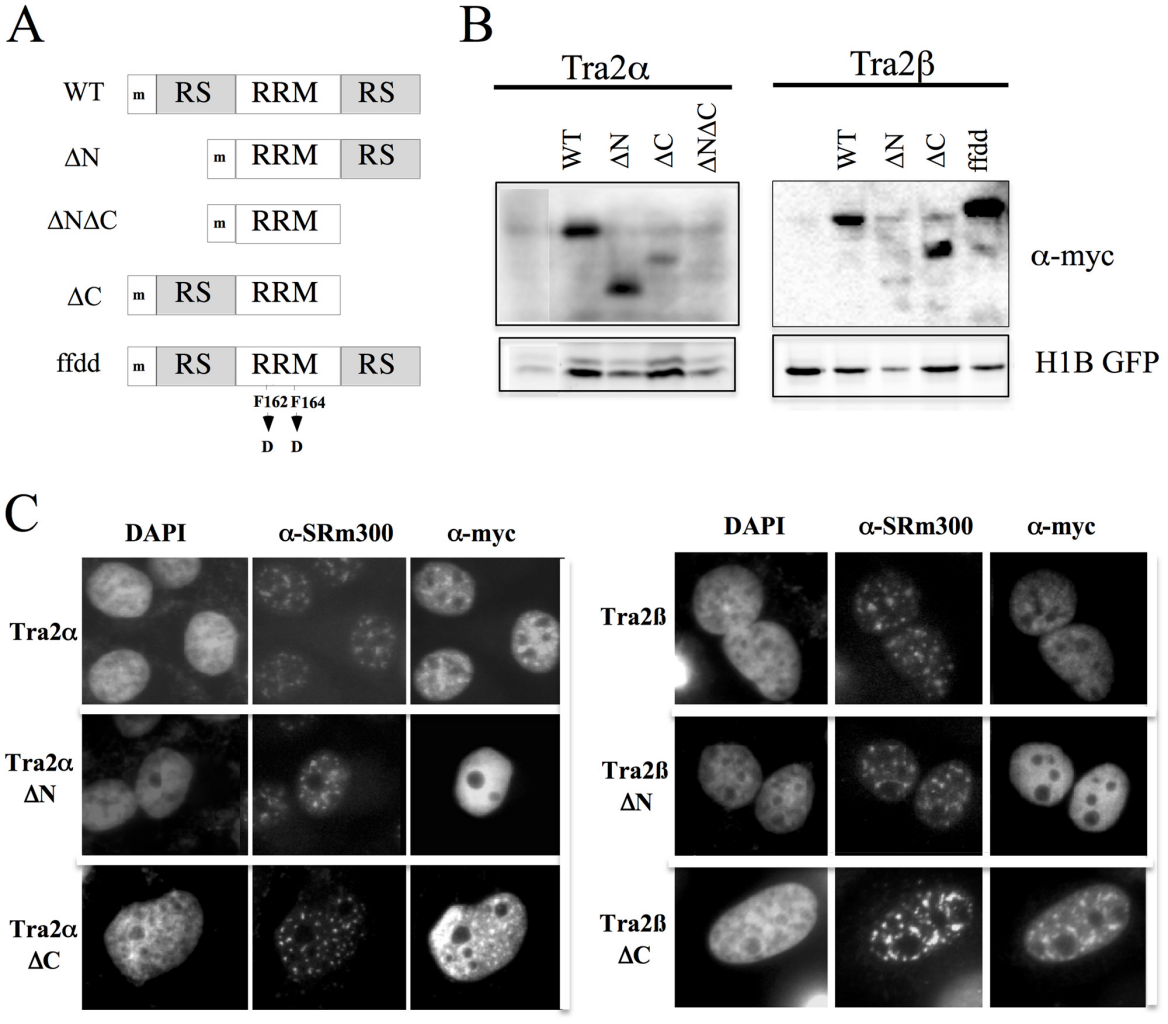
Structure, Expression and Subcellular Distribution of Tra2α/β Mutants. A, Schematic representation of the panel of deletion mutants created for Tra2α and Tra2β. RRM- RNA recognition motif, RS- arginine-serine rich domain, ‘m’ indicates the presence of an N-terminal myc epitope tag. WT refers to the full length protein. B, To examine expression of the Tra2 variants generated, 293T cells were transfected with the myc-epitope tagged constructs indicated as well a plasmid expressing histone 1B fused to GFP (H1B GFP) to normalize for transfection efficiency. Total cell lysate was prepared and fractionated on SDS-PAGE gels. Blots were subsequently probed with anti-myc antibody. C, HeLa cells were transfected on coverslips with CMVmyc Tra2α and mutants thereof (left panel) or CMVmyc Traβ and mutants thereof (right panel). Samples were processed 48 hours post-transfection for the subcellular localization of transfected Tra2α/β proteins and SRm300 as outlined in “Materials and Methods”. Localization of the proteins was determined by indirect immunofluorescence using α-myc antibody and polyclonal α-SRm300 antibody. Position of nuclei was determined by DAPI staining.

In contrast to members of the SR protein family that have only a C-terminal RS domain, Tra2α and Tra2β are distinct, having an RS domain at both termini of the protein separated by an RNA recognition motif (RRM). Given the role of RS domains in mediating protein subcellular distribution and protein-protein interactions (reviewed in [7]), we were interested in examining the role of Tra2α and Tra2β in HIV-1 RNA processing and the contribution of the individual domains in the response observed. To test this point, myc-epitope tagged deletion mutants lacking one or both RS domains were generated (Fig 1A). The Tra2βΔN construct is identical to a natural occurring variant of the protein (designated Tra2β3) [19, 24–26]. Mutation of the RRM (FF to DD) was also performed to investigate the contribution of RNA binding to the activity of the proteins. The impact of the removal of these domains on protein expression, localization and function was then assessed. Western blot of lysates from transfected cells revealed that all but one of the mutants were expressed, the exception being Tra2αΔNΔC which was not detectable above background (Fig 1B).

To assess what effect these domain deletions had on protein localization, we examined their steady state localization following transfection into HeLa cells. As shown in Fig 1C, full length Tra2α is nuclear, non-nucleolar with some accumulation in nuclear speckles (as indicated by co-staining with SRm300), a pattern commonly observed for the SR protein family (reviewed in [38]). Tra2β is also nuclear and non-nucleolar and shows some association with nuclear speckles (Fig 1C). Consistent with recent work by Shu-Jing et al. [39], removal of the N-terminal RS domain from either protein results in a shift to a diffuse nuclear staining, with nucleolar exclusion. While removal of the C-terminal RS domain did not reduce the association of Tra2αΔC with nuclear speckles, the accumulation of Tra2βΔC with the nuclear speckles is enhanced. The different effects of the mutations on subnuclear localization raised the possibility that the N- and C-terminal RS domains may not be equivalent in their activities. However, either RS domain in conjunction with the RRM was sufficient for nuclear accumulation of the proteins.

### Tra2α/β and the ΔN Variants Strongly Suppress HIV-1 Gag and Env Expression

**Fig 2:**
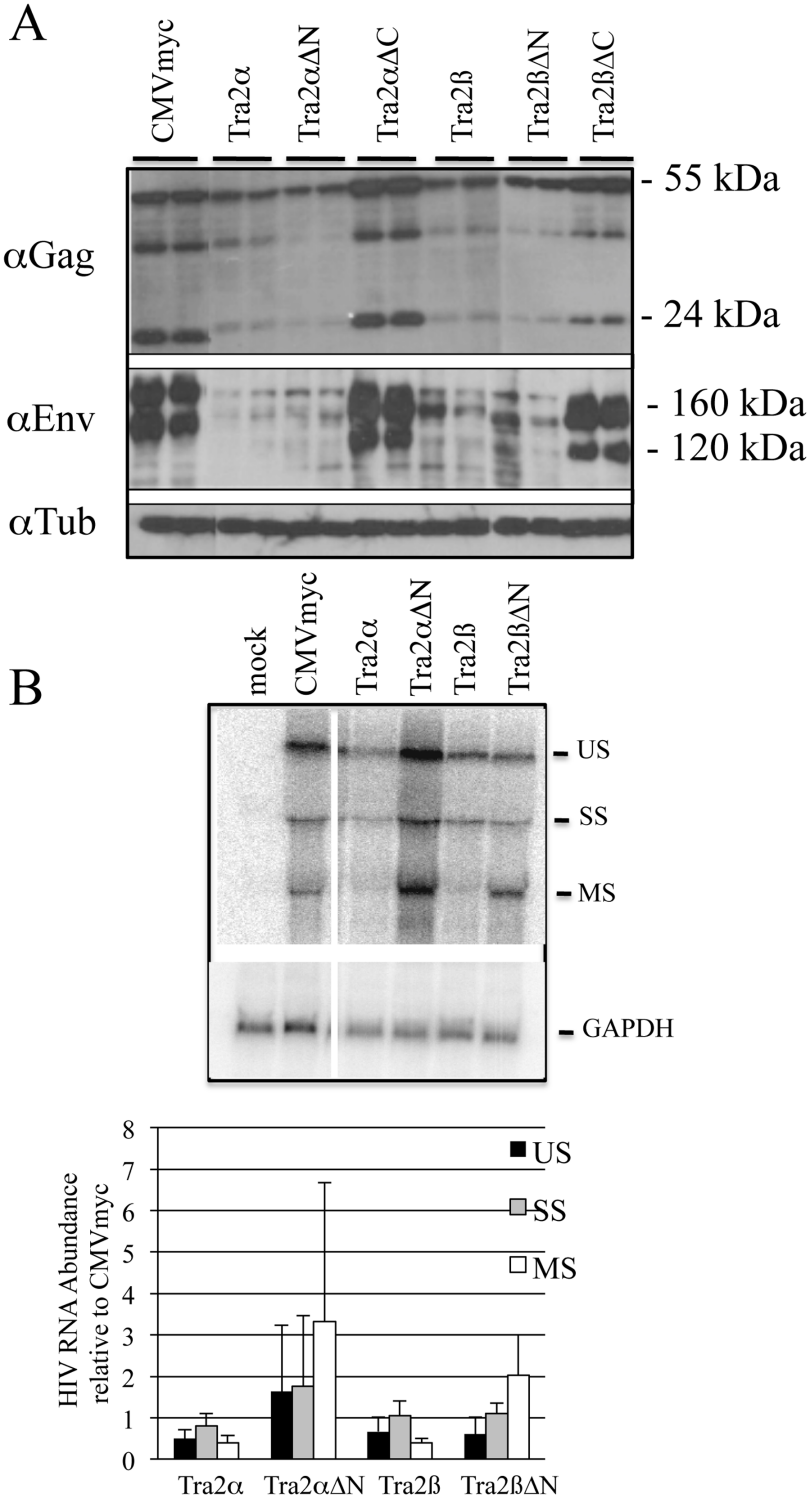
Tra2α/β Overexpression Alters HIV-1 Expression and RNA Accumulation. 293T cells were transfected with pHxb2 R-/RI- and the indicated myc expression plasmids. 48 h post-transfection, cells were harvested for either analysis of (A) HIV-1 Gag and Env expression (loading of blots was confirmed with anti-tubulin antibody) or (B) viral RNA by northern blot. Probes used were specific for HIV-1 or GAPDH RNA. On top is a representative sample of the northern blot results and at bottom, a summary of results of 3 independent assays. Values shown are normalized to the abundance of the respective HIV-1 RNA upon co-transfection with CMVmyc after adjusting for variation in loading using GAPDH RNA.

Having confirmed expression and localization of each of the variants, we examined the impact of their overexpression on HIV-1 gene expression and viral RNA processing. As shown in Fig 2A, western blots of transfected 293T cell extracts revealed that both Tra2α and Tra2β and the N-terminal RS truncations (Tra2αΔN and Tra2βΔN) strongly reduce the level of both HIV-1 Gag and Env proteins relative to control vector (CMVmyc). In contrast, deletion of the C-terminal RS domain of either protein (Tra2αΔC and Tra2βΔC) had little effect. Subsequent northern blots for viral RNA determined that the full length and ΔN variants have different effects on viral RNA levels (Fig 2B). Due to the absence of effect on viral protein expression, analysis of effects of Tra2αΔC and Tra2βΔC on HIV-1 RNA was not performed. Overexpression of either Tra2α or Tra2β had the greatest impact on the abundance of MS HIV-1 RNAs, reducing levels of this RNA to ~40% seen in the control (CMVmyc). In contrast, both Tra2αΔN and Tra2βΔN increased the level of multiply spliced RNAs. The striking difference in effect suggests that the full length and ΔN variants may be functioning in distinct ways.

**Fig 3:**
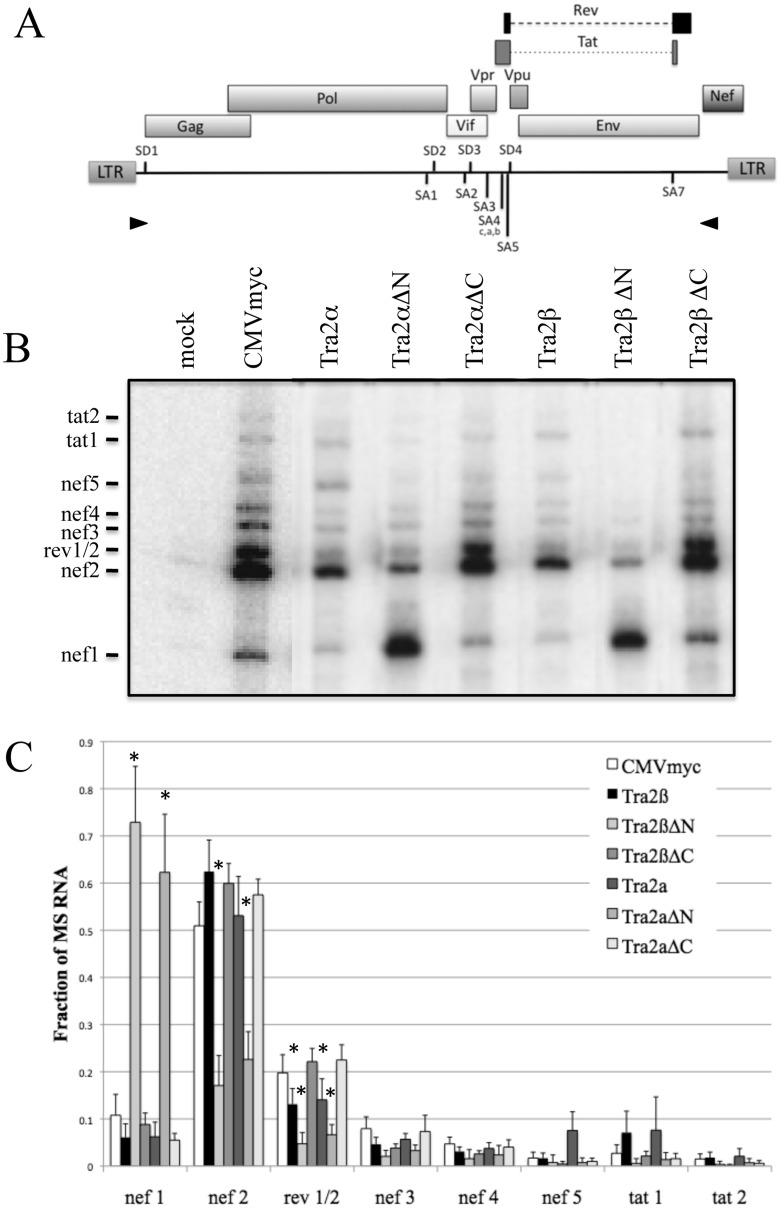
Tra2α/β Overexpression Alters HIV-1 Splice Site Usage. A, Schematic of the HIV-1 provirus, indicating the position of the primers (arrowheads) used for the RT-PCR analysis of HIV-1 MS RNA. B, 293T cells were transfected with pHxb2 R-/RI- with the indicated myc-expression vectors. RNA was extracted, cDNA generated and amplification of HIV-1 MS RNA performed as outlined in “Materials & Methods”. Shown is a representative gel of the results obtained. The products generated are identified on the left. See Fig A in S1 File for a description of the products generated. Products generated from untransfected cells (mock) are also shown. C, Summary of results from n.>3 assays. Values are the relative fraction of total signal for each of the amplicons generated. Asterisks indicated values deemed significant from control (CMVmyc) at p<0.05.

To explore in greater detail how these factors alter HIV-1 RNA processing, we looked for changes in splice site usage by RT—PCR in both the singly and multiply spliced viral RNAs (see Fig A in S1 File) for a listing and description of the spliced products generated by HIV-1). Analysis of singly spliced viral RNAs revealed little or only modest alterations in relative abundance among the different singly spliced RNAs in response to overexpression of any of the proteins (Fig B in S1 File). In contrast, analysis of multiply spliced viral RNAs revealed marked differences (Fig 3). The effects of Tra2α and Tra2β were subtle with the most significant response being a slight reduction in the accumulation of RNAs encoding Rev (rev1/2) (levels are ~70% of control) and increased levels of tat1 RNA. In contrast, Tra2αΔN and Tra2βΔN overexpression induced a dramatic shift, increasing nef1 RNA abundance, corresponding to the ligation of first 5’ss (SD1) with the last 3’ss (SA7). Increased levels of nef1 RNA were associated with reductions in nef2, rev1/2 and tat1 RNAs. Finally, overexpression of Tra2αΔC or Tra2βΔC induced little or no change in splice site use consistent with their failure to alter viral gene expression. Additional tests determined that the response is dependent upon RNA binding as the Tra2βffdd mutant did not alter the pattern of MS RNA splicing relative to control (Fig C in S1 File).

**Fig 4:**
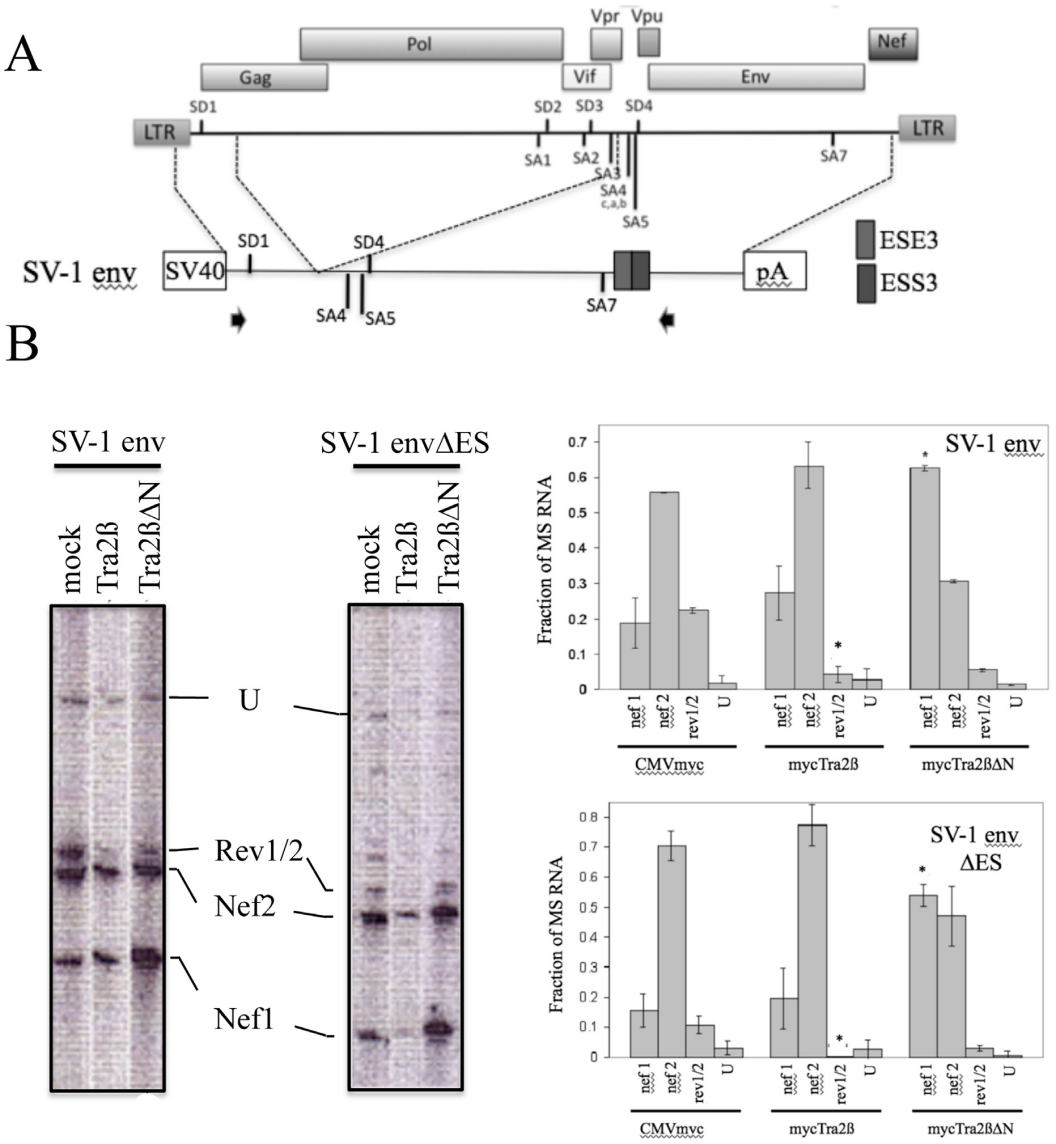
Tra2β Alteration in HIV-1 Splice Site Usage is Independent of ESE3 and ESS3. A, Schematic of the HIV-1 provirus and which viral sequences were used in the generation of the SV-1 env expression vector. Shown is SV40 promoter (SV40), the polyadenylation signal (pA), the position of the splice donors (SD) and splice acceptors (SA) as well as the location of ESE3 and ESS3. SV-1 envΔES was generated by deletion of ESE3 and ESS3. B, 293T cells were transfected with SV-1 env or SV-1 envΔES along with CMVmyc (mock), CMVmyc Tra2β (Tra2β) or CMVmyc Tra2βΔN (Tra2βΔN). RNA was extracted 48h post-transfection and RT-PCR assays performed for HIV-1 MS RNAs as detailed in “Material and Methods”. Shown on the left are representative gels of the RT-PCR products detected and on the right, a summary of the results obtained from n = 3 assays, indicating the relative abundance of a subset of the RT-PCR products detected; U designates unspliced RNA. Asterisks indicated values deemed significant from control (mock) at p<0.05.

The increased levels of MS RNA and the shift to nef1 RNA accumulation in response to overexpression of Tra2αΔN or Tra2βΔN suggested the possibility that these factors were functioning through interaction with one of the ESEs present within HIV-1 RNA. Two of the ESEs (GAR and ESE3) have the GAR-rich motif characteristic of known Tra2β binding sites [27, 40–42]. ESE3 within the terminal exon of HIV-1 regulates recognition of SA7, its inactivation resulting in loss of SA7 use due to the presence of an adjacent exon splicing silencer (ESS3) [43, 44]. To assess whether the responses to either Tra2β or Tra2βΔN may be mediated through ESE3, we examined the effect of its mutation on the pattern of splice site use. To facilitate analysis, the assays were performed in the context of the SV-1 env vector [30, 45] which retains the SD1, SA4a,b,c, SA5, SD4 and SA7 HIV-1 splice sites by deletion of the Gagpol, Vif, and Vpr reading frames (Fig 4A). SV-1 env was cotransfected with control vector (CMVmyc), Tra2β or Tra2βΔN expression vectors, RNA isolated and analyzed by RT-PCR. As shown in Fig 4B, overexpression of Tra2β reduced rev1/2 RNA accumulation while Tra2βΔN increased nef1 RNA levels, consistent with effects seen using the complete provirus. Analysis in the context of SV-1 env ΔES (deleting ESS3 and ESE3 within the terminal HIV-1 exon) revealed a similar pattern of response upon Tra2β or Tra2βΔN overexpression (Fig 4B), indicating that neither element mediates the observed responses to these factors.

### Effect of Tra2β Variants on HIV-1 US RNA Localization

**Fig 5:**
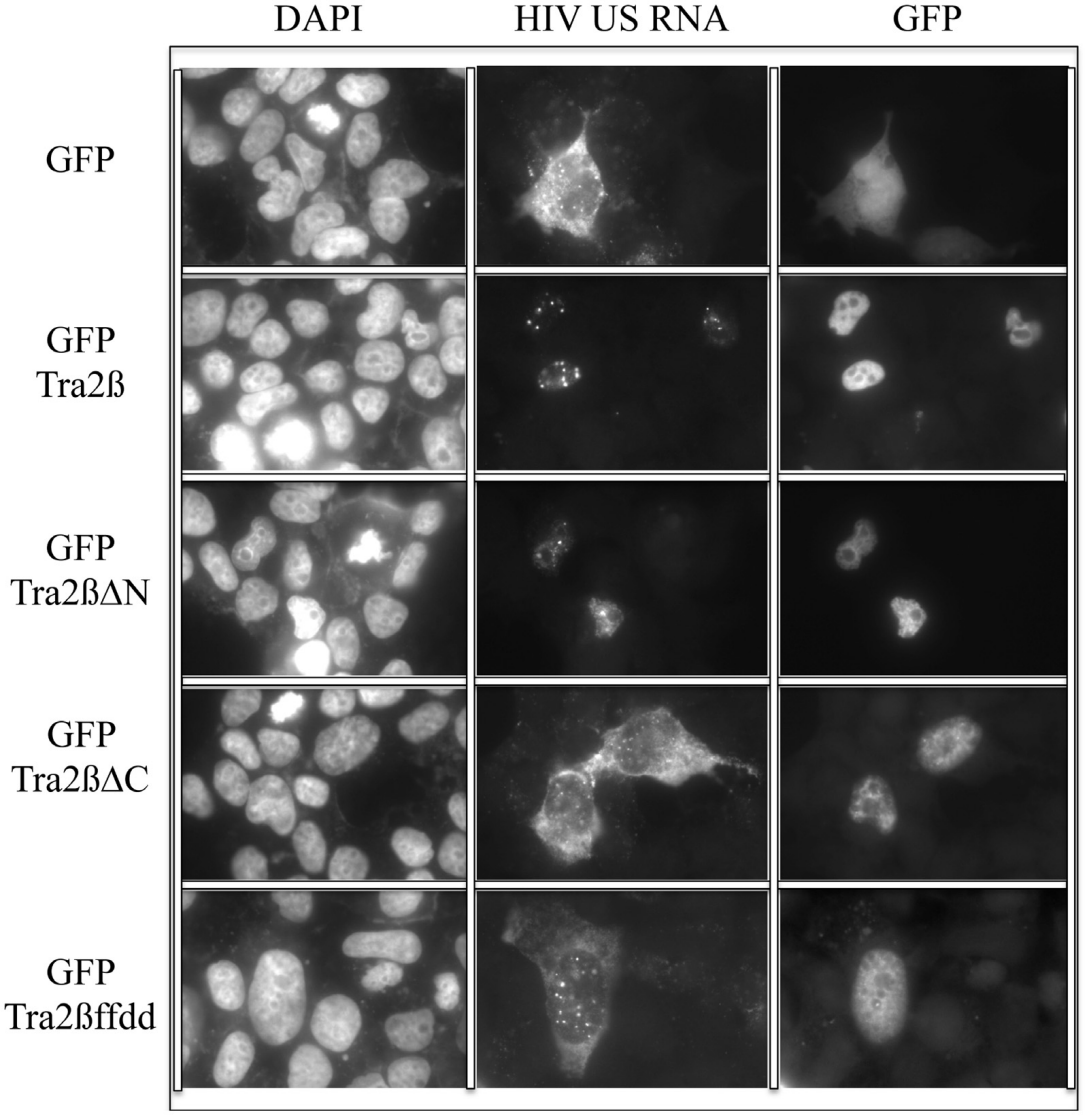
Modulation of HIV-1 US RNA Transport by Tra2β/βΔN Overexpression. 293 cells were transfected with pHxb2 R-/RI- and plasmids expressing GFP (GFP) or GFP—tagged Tra2β (GFP Tra2β), Tra2βΔN (GFP Tra2βΔN), Tra2βΔC (GFP Tra2βΔC), or Tra2βffdd (GFP Tra2βffdd). Cells were fixed 48 h post-transfection and processed for localization of HIV-1 US RNA and GFP-tagged protein (GFP) as detailed in “Materials and Methods”. Nuclei were detected by staining with DAPI.

**Fig 6:**
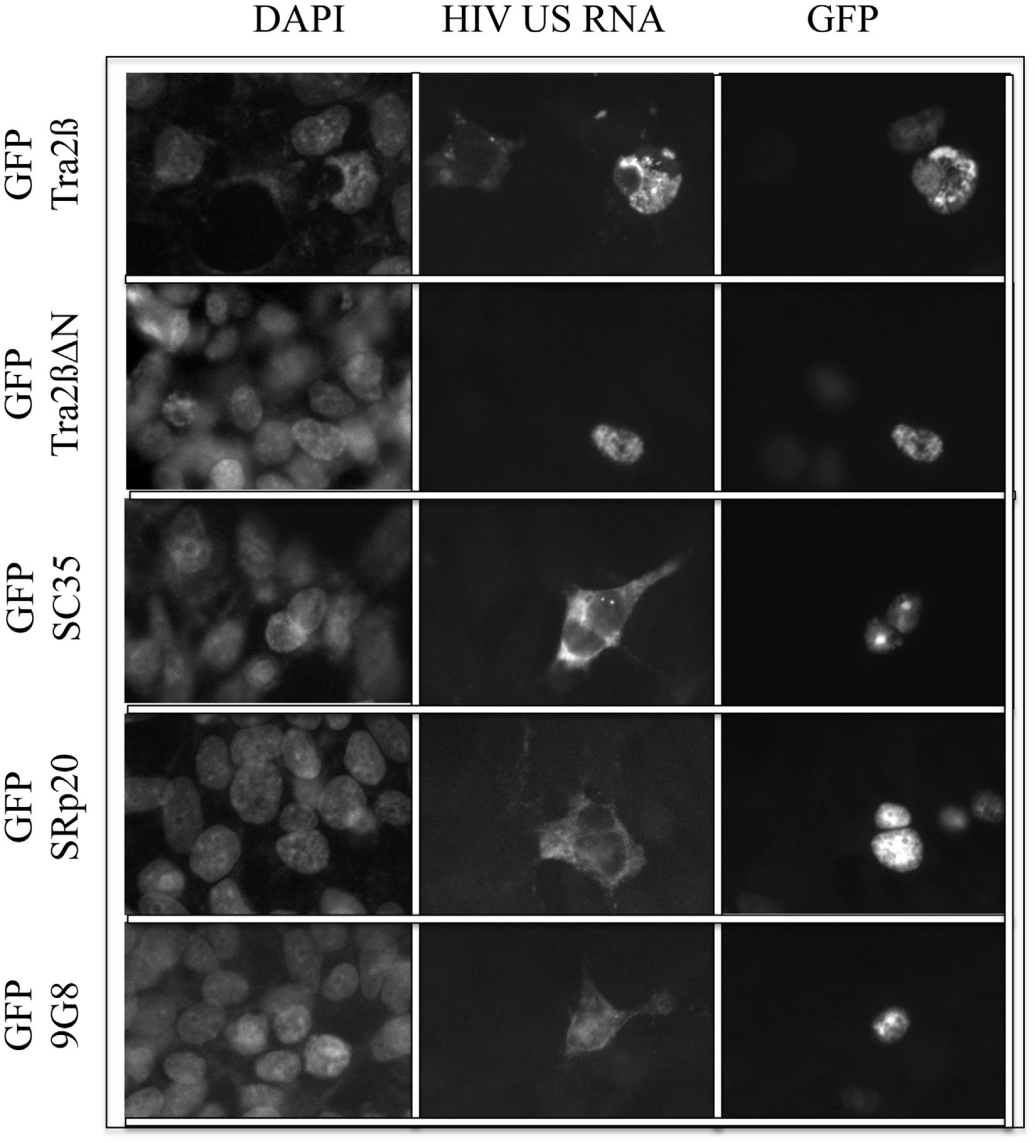
Nuclear Sequestration of HIV-1 US RNA does not occur upon overexpression of other SR proteins. 293 cells were transfected with pHxb2 R-/RI- and plasmids expressing GFP (GFP) or GFP—tagged Tra2β (GFP Tra2β), Tra2βΔN (GFP Tra2βΔN), SC35 (GFP SC35), SRp20 (GFP SRp20), or 9G8 (GFP 9G8). Cells were fixed 48 h post-transfection and processed for localization of HIV-1 US RNA and GFP-tagged protein (GFP) as detailed in “Materials and Methods”. Nuclei were detected by staining with DAPI.

The absence of HIV-1 Gag and Env expression in the presence of either Tra2β or Tra2βΔN, despite the presence of significant levels of the corresponding viral (US and SS) RNAs (Fig 2B), suggested the possibility that these factors might act to alter viral RNA transport. To address this hypothesis, in situ hybridization was used to examine viral US RNA subcellular localization in response to overexpression of Tra2β or its variants. As shown in Fig 5, co-transfection of HIV-1 proviral DNA with control vector (peGFP) resulted in the majority of the US viral RNA being localized to the cytoplasm. A similar pattern of HIV-1 US RNA distribution was also observed upon co-transfection with Tra2βΔC or Tra2βffdd, neither of which altered HIV-1 RNA processing (Fig 3 and Fig C in S1 File). In contrast, co-transfection with either Tra2β or Tra2βΔN resulted in the viral US RNA being present predominately in the nucleus, indicative of a block to nuclear export. Similar overexpression studies using GFP fused to SC35, SRp20 or 9G8 did not elicit a similar nuclear sequestration of HIV-1 US RNA (Fig 6), indicating that the response to Tra2β/Tra2βΔN overexpression is not a general response to SR overexpression but is limited to a least a subset of proteins within the SR family. Additional tests (Fig D in S1 File) confirmed expression and the effect of the GFP fusion proteins on HIV-1 gene expression.

**Fig 7:**
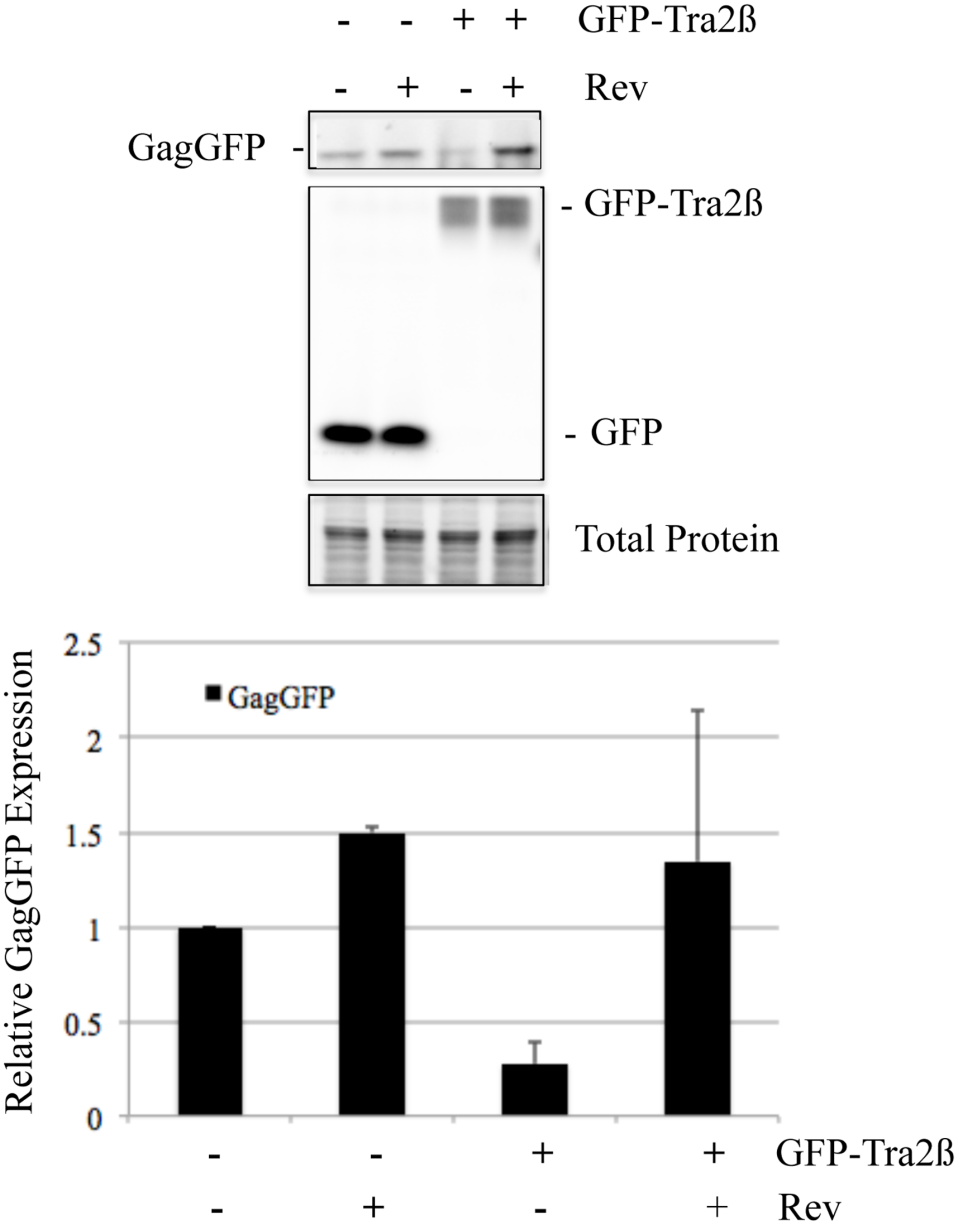
Rev Overexpression Reverses the Repression of HIV-1 Gag by Tra2β. HEK 293T cells were transfected with HIV-1 proviral clone pNL4-3 GagzipGFP in the presence of either plasmid expressing GFP or GFP-Tra2β with (+) or without (-) a Rev-expression vector. Forty-eight hours post transfection, cells were harvested and extracts analyzed on SDS-PAGE gels. GFP signals for GagGFP, GFP, and GFP-Tra2β were imaged directly using a Bio-Rad MP imager. Total protein loading was verified using Stain-Free gels (Bio-Rad). At top is a representative assay and bottom, a summary of results from n>3 independent assays.

To explore possible mechanisms by which Tra2β induced nuclear retention of HIV-1 US RNA, we tested whether Rev overexpression could rescue viral Gag expression. As shown in Fig 7, cotransfection of a HIV-1 proviral construct with a vector expressing GFP should little difference upon Rev overexpression. In contrast, overexpression of GFP-Tra2β significantly decreased Gag expression and its effect was reversed upon Rev overexpression.

### Effect of Tra2β Depletion on HIV-1 Protein and RNA Expression

**Fig 8:**
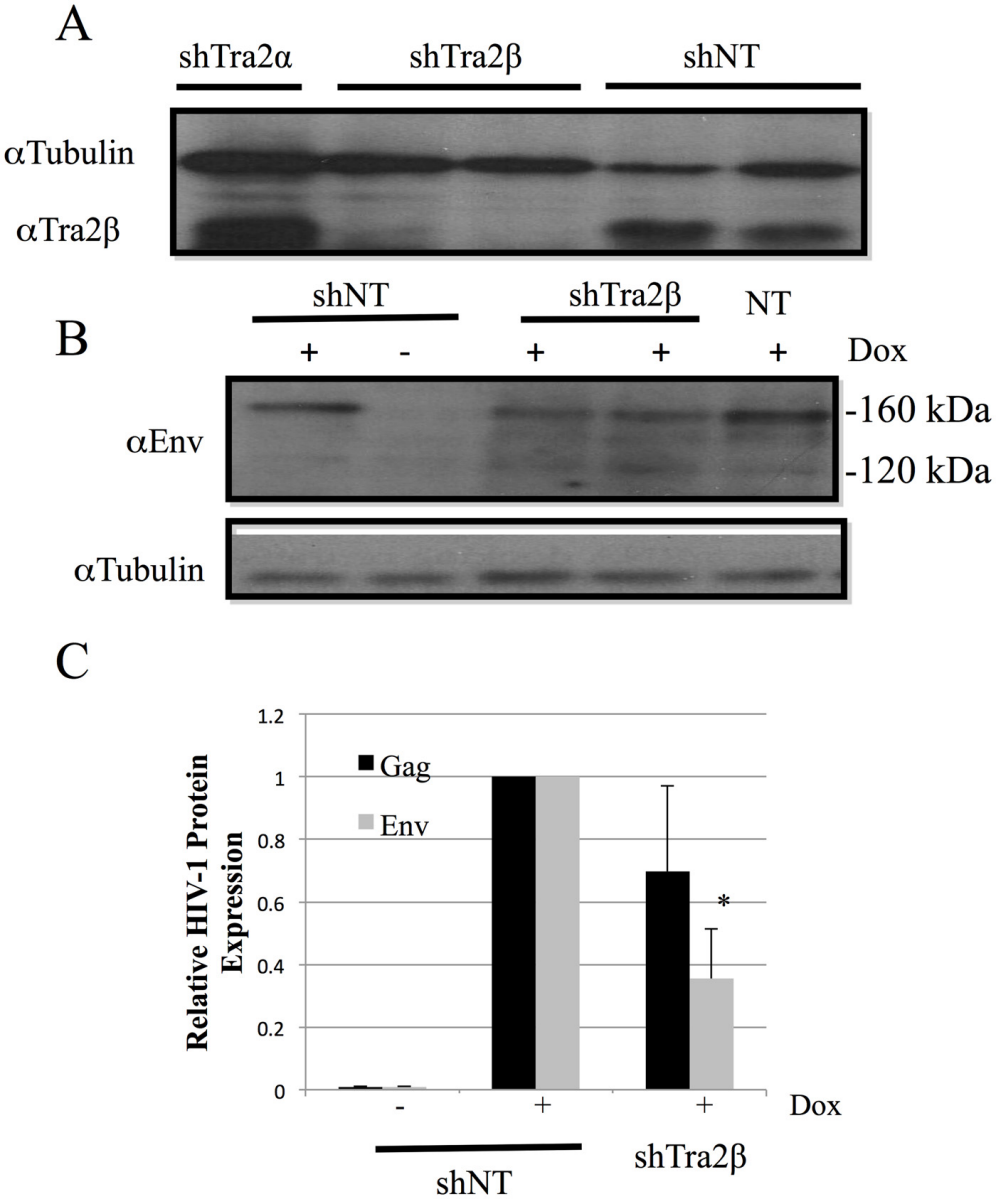
Tra2β Depletion Reduces HIV-1 Env Protein Accumulation. Cells containing an integrated, doxycycline-inducible HIV-1 provirus (HeLa rtTA HIVΔmls) were transduced with lentiviruses expressing either control (shNT) or shRNA targeting Tra2β(shTra2β) or Tra2α (shTra2α) and a cassette conferring puromycin resistance. Transduced cells were selected using puromycin for 48–72 h, then HIV-1 provirus expression induced by addition of doxycycline. Cell extracts were subsequently analyzed by western blot to (A) confirm Tra2β depletion, (B) examine alterations in HIV-1 Env expression by western blot, or (C) for changes in HIV-1 Gag levels by p24 ELISA. Shown is a summary of results obtained of n>3 assays. Asterisks indicated values deemed significant from control (shNT) at p<0.05.

**Fig 9:**
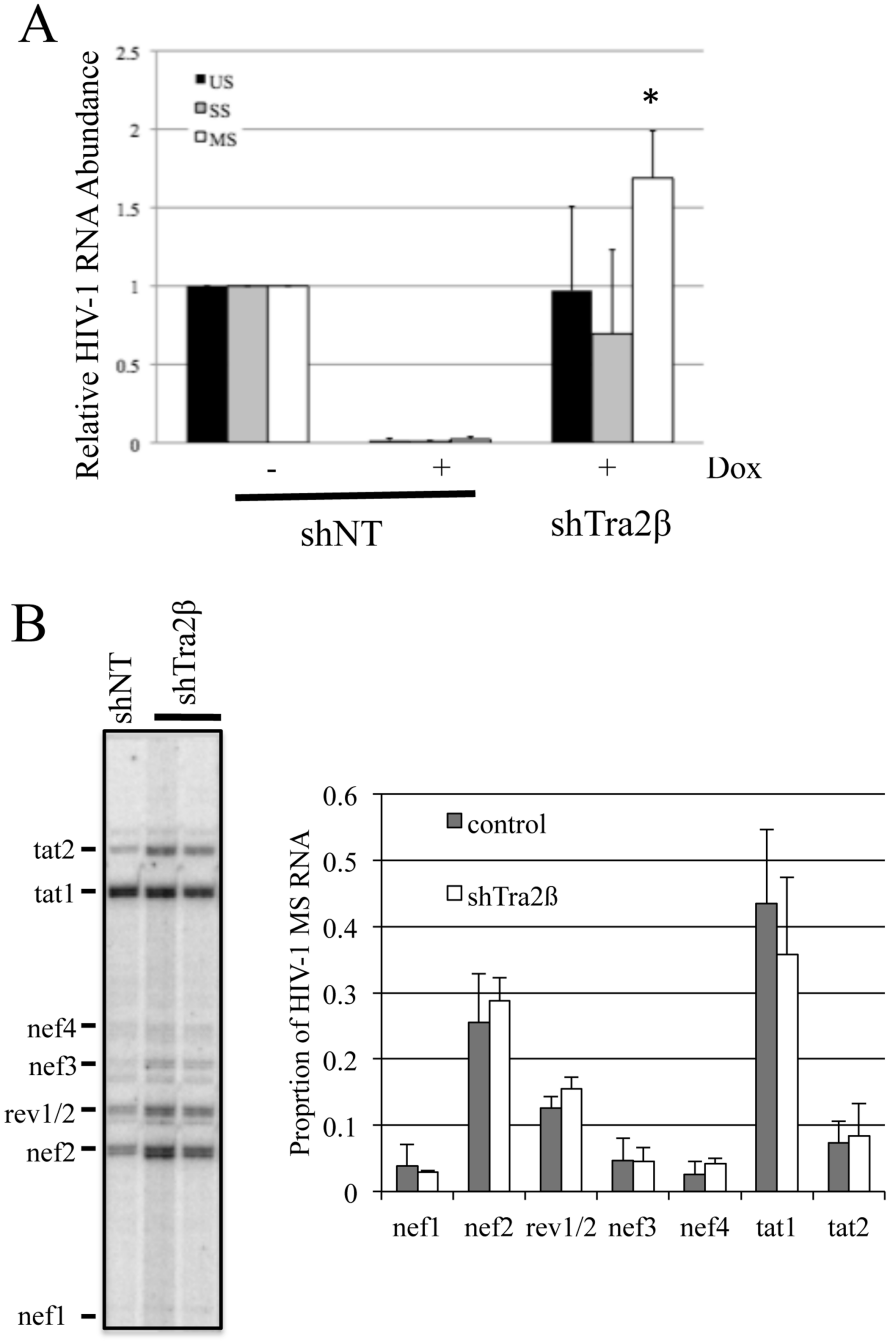
Tra2β Depletion Results in Selective Alterations in HIV-1 RNA Accumulation. HeLa rtTA HIVΔmls cells were transduced with lentiviruses expressing either control (shNT) or shRNA targeting Tra2β (shTra2β). After infection, transduced cells were selected using puromycin for 48–72 h, then HIV-1 provirus expression induced by addition of doxycycline. RNA was subsequently extracted and analyzed (A) by qRT-PCR to examine for changes in HIV-1 US, SS and MS RNA levels or (B) RT-PCR of MS RNAs to examine for changes in splice site usage. Asterisks indicated values deemed significant from control (shNT) at p<0.05.

To complement the above overexpression studies and explore the role of the endogenous Tra2β in the regulation of HIV-1 RNA processing, targeted depletion studies were performed. To facilitate analysis, depletion experiments were performed in the context of the HeLa rtTA HIVΔmls cell line [37]. This cell line contains stably integrated, doxycycline (Dox) inducible HIV-1 provirus [46, 47]. which has a deletion of the reverse transcriptase and integrase reading frames to render it unable to replicate. Recent studies by our group have shown that overexpression of either Tra2β or Tra2βΔN in this cell line yielded similar alterations in HIV-1 RNA splice site selection as seen upon introduction into 293/293T cells used in this study [48]. To deplete Tra2β, cells were infected with lentivirus expressing control or shRNA to Tra2βand transduced cells isolated by treatment with puromycin. After puromycin selection for transduced cells, HIV-1 provirus expression was induced by addition of Dox for 24 h following which media and cells were harvested to analyze for changes in HIV-1 protein and RNA expression. As indicated in Fig 8A, significant depletion of Tra2β was achieved under the conditions used. Subsequent evaluation of HIV-1 Gag and Env protein levels revealed Tra2β depletion reduced Env synthesis slightly (~2 fold) (Fig 8B and 8C) but had a limited effect on Gag expression (Fig 8C). In light of these observations, we looked at whether these manipulations induced any changes in viral RNA abundance. qRTPCR analysis of HIV-1 RNA abundance determined that reduction of Tra2β levels resulted in a selective increase in MS RNA levels with no significant effect on US or SS RNA accumulation (Fig 9A). Parallel analysis of the MS RNA splicing pattern (Fig 9B) determined that there was no significant alteration in splice site use within this viral RNA class upon Tra2ß depletion.

## Discussion

Studies to date have highlighted a significant role for SR proteins in regulating HIV-1 RNA processing and expression, different members of the SR protein family yielding distinct effects upon either overexpression or depletion [30, 48–51]. In general, these factors have been thought to act via the interaction with specific ESEs within the HIV-1 RNA to augment recognition of adjacent weak splice sites and/or to block the action of adjacent ESSs by steric inhibition of hnRNP A1 binding [4]. Recent studies by Erkelenz *et al*. [52] have demonstrated a role for Tra2α/β in the modulation of HIV-1 RNA splicing through interaction with an ESE (ESE_vpr_) adjacent to SA2, used in the expression of Vpr. As an extension of these studies, we report here on the impact of overexpression and depletion of full length Tra2α/β as well as on the activity of variants thereof on HIV-1 RNA processing, transport, and expression.

Overexpression of Tra2α/β yielded a marked reduction in HIV-1 Gag and Env expression which was accompanied by some changes in viral RNA abundance and alterations in the pattern of viral RNA splicing. The changes in viral RNA upon overexpression of Tra2α/β were most evident in reduced MS RNA accumulation as well as a trend to reduced US RNA abundance. Levels of SS RNA were largely unaffected. In contrast to the report by Erkelenz *et al*. [52], our examination of the splicing within the HIV-1 SS RNAs did not detect significant alterations upon Tra2α/β overexpression. In the study by Erkelenz *et al*., Tra2α/β overexpression enhanced usage of SA2, resulting in increased levels of vpr, tat3, env8, and nef4 RNAs, all containing HIV-1 exon 3. Differences between the two studies may be attributable to the virus strains used (NL4-3 versus HxB2). Analysis of MS RNA splicing patterns revealed a slight reduction of rev1/2 RNA (~70% of control) upon Tra2α/β overexpression. Such a small reduction would not be expected to result in significant changes in Rev protein that is essential for HIV-1 Gag and Env protein expression. The change in viral protein expression could be attributed to the effect on HIV-1 RNA localization, as overexpression of Tra2β resulted in nuclear retention of viral US RNA. This response is somewhat unique to Tra2β as overexpression of other SR proteins (SC35, SRp20, 9G8) did not have a similar effect and contrasts with the role of SRp20 and 9G8 in promoting RNA export in other systems [53, 54]. One hypothesis for the response observed is that overexpression of Tra2β accelerates the commitment of HIV-1 RNA to splicing and nuclear sequestration. Previous studies by our group have shown that the activity of some nuclear retention sequence (NRS) elements requires the presence of a 5’ splice site (5’ss) [55]. In the case of HIV-1, the activity of 5’ss SD4 is dependent upon the presence of a purine rich ESE (designated GAR), inactivation of which leads to dramatic alterations in HIV-1 RNA processing as evidenced by increased nef1 RNA accumulation [30, 45]. Previously, we have demonstrated that HIV-1 RNA already committed to splicing/nuclear retention is not available for transport by Rev [56]. Overexpression of Tra2β, by enhancing SD4 recognition, may increase the rate at which HIV-1 US RNA becomes committed to splicing/nuclear sequestration. Consistent with this hypothesis, we observed that overexpression of Rev restored expression of HIV-1 Gag (Fig 7) indicating that Tra2β does not directly interfere with Rev function.

Of particular note was our determination that variants of Tra2α/β lacking one of the RS domains had strikingly different activities in the modulation of HIV-1 processing from the full length protein and each other, a result that was recapitulated upon comparison of these variants in another context, that of the doublesex RNA splicing cassette (see Fig E in S1 File). Previous studies examining differences in RS domain activity among various SR proteins determined that they were functionally interchangeable and, in some instances, varied in activity by no more than 2–3 fold [19, 57–60]. RS domains affect RNA splicing either by mediating protein-protein interactions [61] or promoting the formation of RNA duplexes [62] such as those between U snRNAs and the branchpoint sequence. RS domain activity is correlated with the number of RSRS repeats, variants having a higher number of these repeats displaying increased activity *in vitro* [58]. In particular, tests using the N- and C-terminal RS domains of Tra2α indicated that they had low but equivalent activity when used to replace the RS region of SF2/ASF [57] and, in the context of SMN alternative splicing, deletion of either RS domain of Tra2β led to an equivalent reduction in activity [63]. Consequently, it was unexpected that the Tra2βΔN would have such potent activity in the context of HIV-1 (and the doublesex) RNA processing while Tra2βΔC was inactive. Our functional tests of the individual Tra2β RS domains determined that both were functional (Fig F in S1 File). Some of the differences between Tra2βΔN and Tra2βΔC could be attributed in part to the difference in localization of each factor; Tra2βΔN showing diffuse nuclear staining while Tra2βΔC displayed some localization to nuclear speckles in agreement with the recent work of Shu-Jing et al. [39]. However, particularly striking was the distinct activity of Tra2βΔN versus Tra2β on HIV-1 RNA processing. We found in the case of HIV-1, overexpression of Tra2βΔN increased the level of MS RNA, and shifted splicing to nef1. Initial studies of the naturally occurring form of Tra2βΔN (designated Tra2ß3) found it to be inactive in the assay system used [26] suggesting that alteration in Tra2β RNA splicing to promote Tra2β3 expression would be equivalent to depletion of full length Tra2β. However, more recent studies [64] have determined that, for a number of alternative exons, overexpression of Tra2β1 and Tra2β3 have opposing activities, the former promoting exon inclusion while the latter induces exon exclusion. Consequently, this study along with our analyses would suggest that a shift in relative abundance of full length (Tra2β1) versus Tra2β3 would cause dramatic alterations in splicing of target RNAs. Tests indicate that the dominate form expressed in the cell lines used in this study is the full length Tra2β1 (data not shown) but work by Nayler *et al*. has shown that the ratio of Tra2β1 to Tra2β3 expression varies in a tissue specific manner [25]. However, the basis for the difference in activity of Tra2β, Tra2βΔC, and Tra2βΔN remains unclear. Given that these factors share the same RRM, the major contributor to RNA binding [63, 65], it is anticipated that they would bind to the same sites within the affected RNA. Consequently, it could be that the difference in activity of Tra2β, Tra2βΔC, and Tra2βΔN would not be due to differences in where on the RNA the factors bound but the interactions each factor mediated at the site. Alternatively, these factors could be altering HIV-1 RNA processing indirectly by changing the expression of other host factors. Consistent with this hypothesis, recent CLIP mapping of Tra2β interaction sites in mouse cells determined that it binds, at high frequency, to genes linked with RNA post-transcriptional modification [64]. However, if they are acting via direct interaction with HIV-1 RNA, the shift in splice site usage with HIV-1 MS RNAs upon overexpression of either Tra2β or Tra2βΔN is not attributable to interactions with either ESE3 or ESS3 within the terminal exon of HIV-1 as deletion of both elements did not alter the ability of these factors to induce changes in splice site usage. Furthermore, since the SV-1 env constructs lack the region encompassing ESE_vpr_ (the recently mapped site for Tra2β interaction [52]), the effects observed cannot be attributed to interaction with this element. However, the shift in HIV-1 splice site use observed upon Tra2βΔN overexpression is comparable to that seen upon mutational inactivation of the GAR ESE 5’ of the SD4 splice site [45]. This similarity suggests the possibility that binding of Tra2βΔN blocks the function of the GAR ESE by either binding to this region of HIV-1 RNA, blocking binding of endogenous factors, or altering the activity/expression of the factors (SF2/ASF and SRp40) known to bind this sequence [45]. If Tra2βΔN acted solely by competitively inhibiting the action of endogenous proteins, one would anticipate that protein variants capable of binding the target RNA sequence would be equally active. However, given the difference in activity of Tra2βΔN and Tra2βΔC, either the N- and C-terminal RS domains differentially affect the ability of the RRM to bind RNA or they mediate different protein-protein interactions.

Additional evidence in support of the important role that Tra2β plays in regulating HIV-1 RNA processing was the finding that its depletion also resulted in changes in viral protein and RNA accumulation. An effect was observed despite the continue presence of Tra2α which could buffer the effect of Tra2β depletion on HIV-1 RNA processing. However, that a response was detected suggests that Tra2β may have a distinct role in regulating viral RNA metabolism. Unlike overexpression, depletion of Tra2β was associated with an increase in HIV-1 MS RNA abundance and a decrease in Env expression. Only very limited effects were observed on US and SS RNA abundance or Gag accumulation as well as no significant changes in the pattern of splice site use within the MS RNAs possibly due to endogenous Tra2α or other factors with overlapping binding specificities substituting for the depleted Tra2β in splice site regulation. Given that Tra2β overexpression reduced MS RNA levels, our findings suggest that Tra2β has an important role in regulating the abundance of HIV-1 MS RNA. One explanation for the observed responses is that Tra2β acts to promote the processing of SS RNA to MS RNA, possibly by competing with or modulating the activity of factors interacting with the GAR ESE to regulate formation of spliceosomes using SD4 and SA7. This activity is clearly dependent on the presence of both RS domains as deletion of either resulted in a marked alteration in activity.

Together, these findings provide insights into the important role that Tra2β plays in regulating HIV-1 RNA processing, in particular the demonstration that the different isoforms of Tra2β (Tra2β1 and Tra2β3) are potent regulators of HIV-1 expression but through distinct effects on viral RNA processing. Recent studies by our group and others [48, 66] have demonstrated that the extent of Tra2β modification can be altered by treatment with digoxin, a response that is associated with a mark inhibition of HIV-1 gene expression and replication [48]. Consequently, understanding how the activity of Tra2β is regulated (either through modification or alteration in isoform abundance) offers the possibility that its activity could be modulated to generate a state in the cell which is unable to support HIV-1 replication through changes in viral RNA processing.

## Supporting Information

S1 File (PDF)

S2 File (DOCX)
