# Supplementary figures and images for: Differential Effects of Tra2ß Isoforms on HIV-1 RNA Processing and Expression
Source: PLoS One. 2015 May 13;10(5):e0125315. doi: 10.1371/journal.pone.0125315 (PMC4430212; doi:10.1371/journal.pone.0125315)

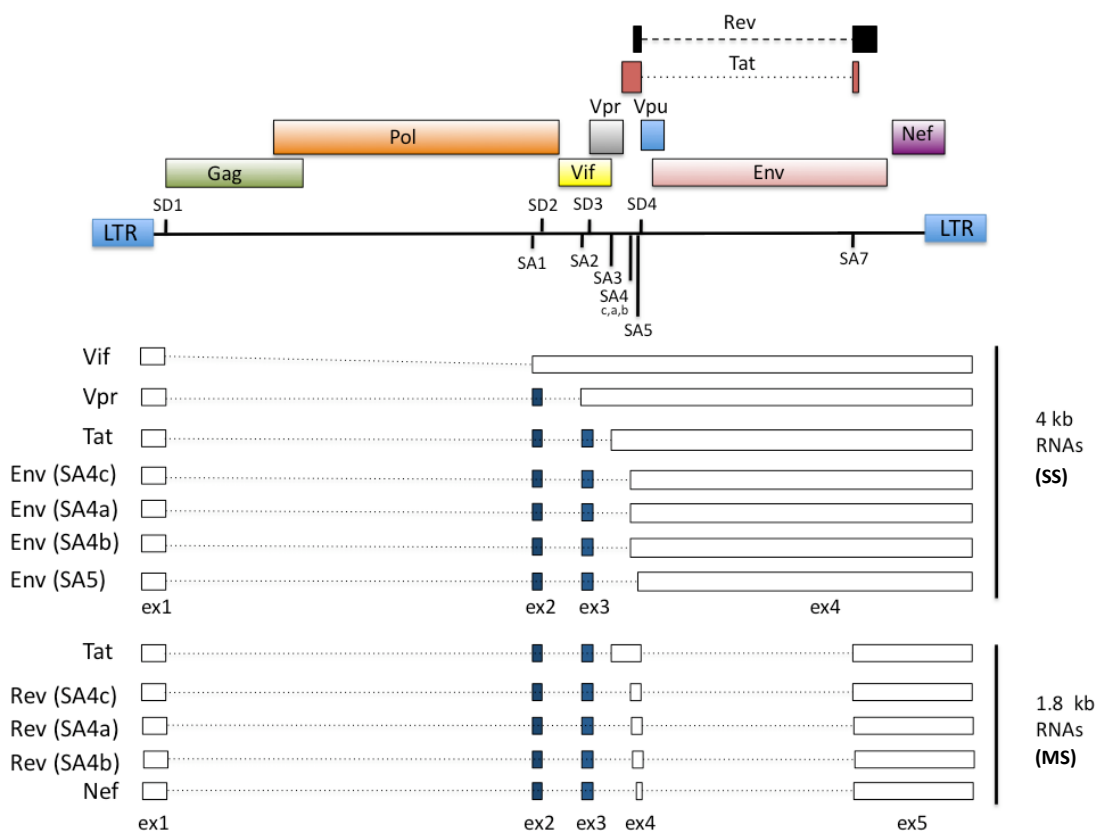

Figure A

A

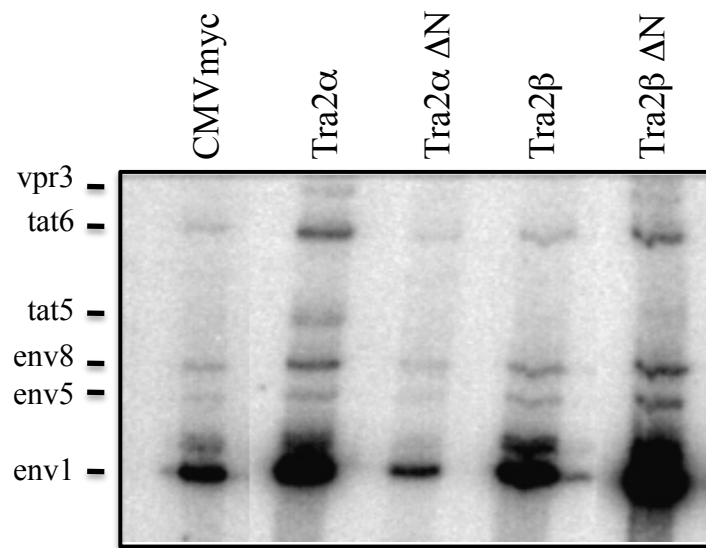

B

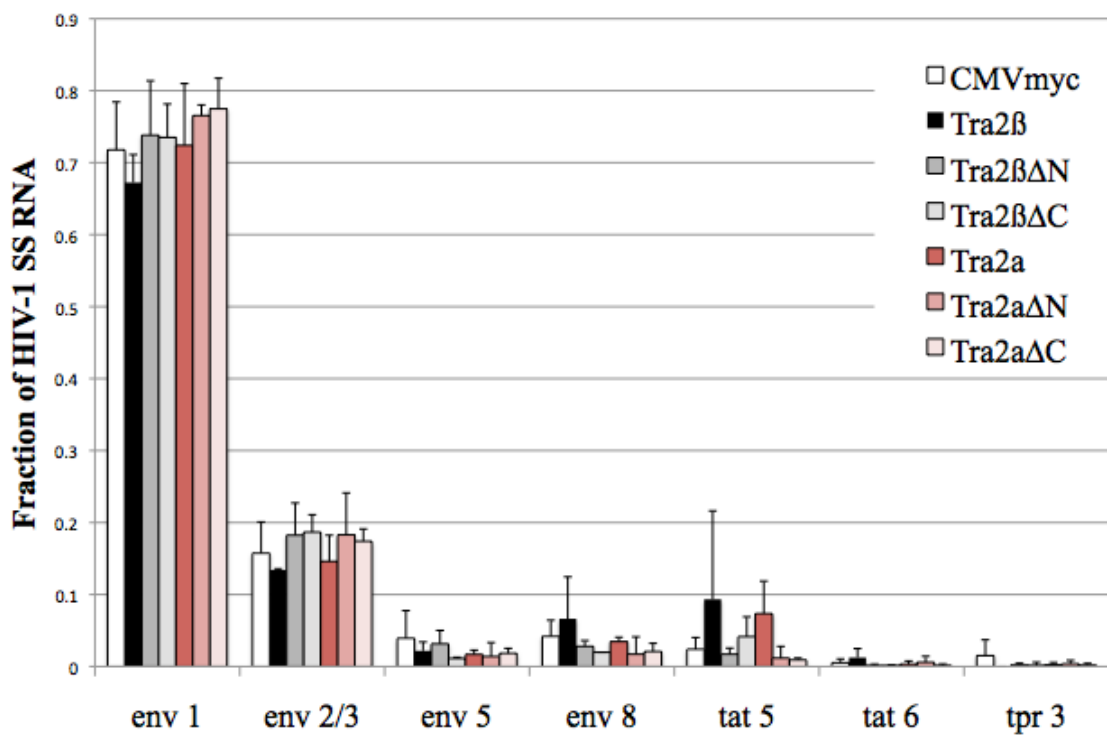

Figure B

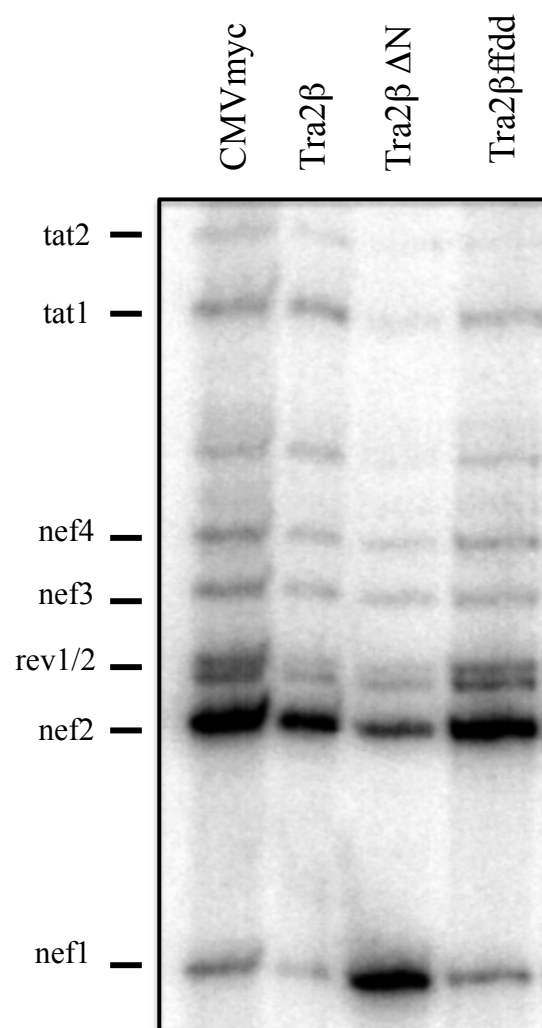

Figure C

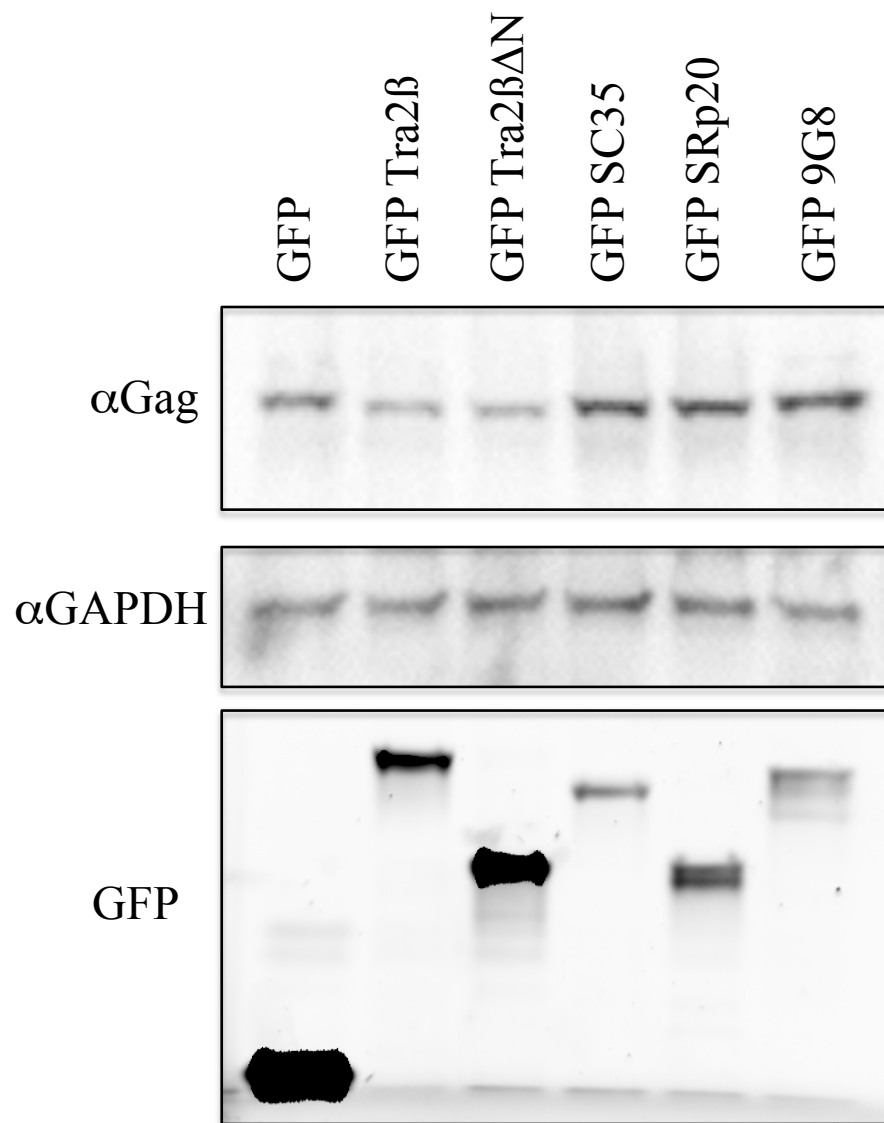

Figure D

A

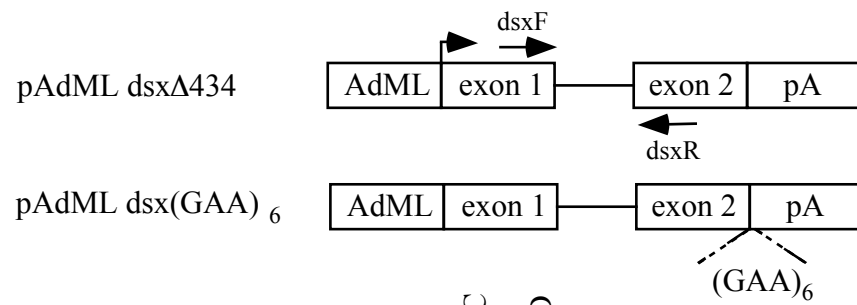

B

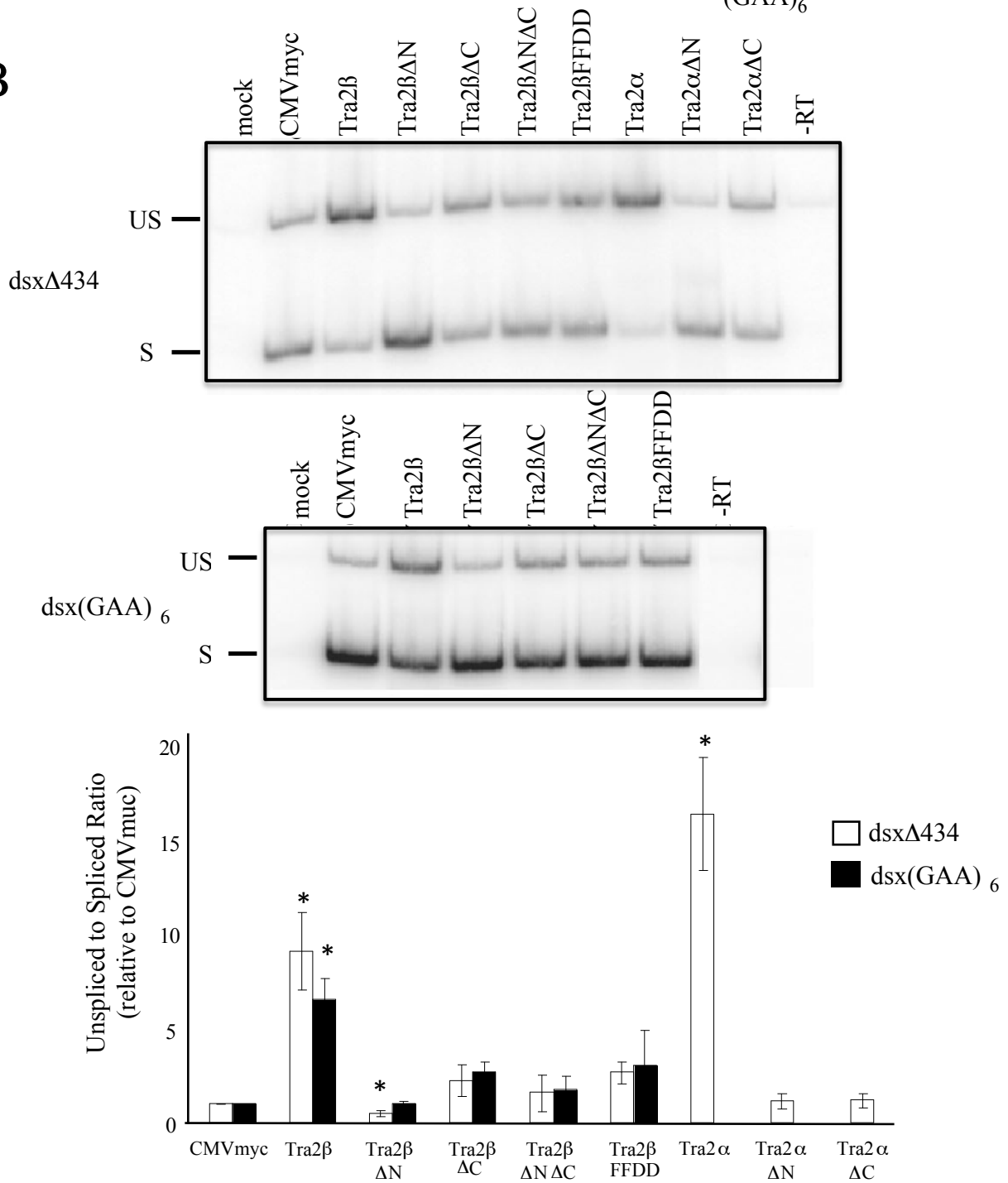

Figure E

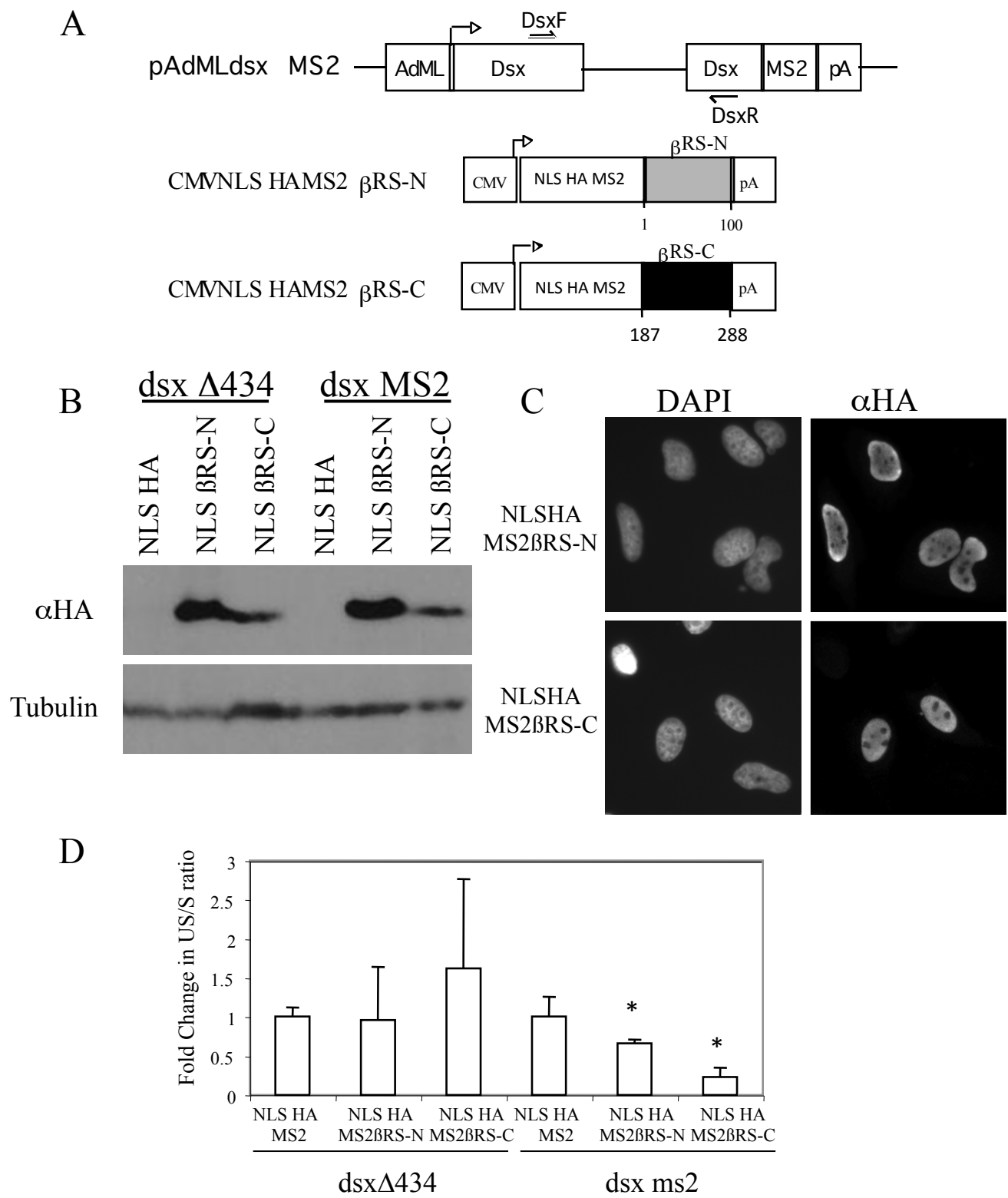

Figure F

Supplement: S1 File — (PDF) [file pone.0125315.s001.pdf]
