# Supplementary material for: Differential Effects of Tra2ß Isoforms on HIV-1 RNA Processing and Expression
Source: PLoS One. 2015 May 13;10(5):e0125315. doi: 10.1371/journal.pone.0125315 (PMC4430212; doi:10.1371/journal.pone.0125315)
Supplement: S2 File — (DOCX) [file pone.0125315.s002.docx]

**Supplementary Results**

**Tra2α and Tra2βMutants Also Differ in the Modulation of dsx RNA Splicing**

The marked difference in activity between the variants of Tra2α and Tra2β (WT, ∆N and ∆C) in the context of HIV-1 was unexpected. Consequently, we were interested in examining whether they displayed similar functional differences in modulating the splicing of another target RNA. As a test, we co-transfected Tra2α/Tra2β expression vectors with a construct comprised of the Drosophila *doublesex* (dsx) gene exons 3 and 4 as well as the intervening intron with (dsx(GAA)_6_) or without (dsx∆434) an artificial, purine-rich ESE known to mediate Tra2β function (Fig. E in File S1). It was anticipated that overexpression of either Tra2α/Tra2β would enhance dsx RNA splicing in an ESE-dependent manner. However, in contrast to expectations, both human Tra2α and Tra2β induced accumulation of unspliced dsx RNA (Fig. E in File S1). A similar response was observed with the dsx∆434 construct, indicating that the effect is independent of the purine-rich ESE (Fig. E in File S1). Overexpression of constructs lacking the N-terminal RS domain (Traα/β∆N) (Fig. E in File S1) enhanced splicing in the context of the dsx∆434 substrate (a 2 fold increase (p<0.02) in the unspliced/spliced dsx RNA ratio) Subsequent analysis determined that the responses observed upon Tra2β and Tra2β∆N overexpression were proportional to the level of transfected plasmid (data not shown). Enhancement of dsx RNA splicing was not observed upon Tra2α∆N and Tra2β∆N expression in the context of dsx(GAA) _6_, possibly due to the increased efficiency of splicing in this construct. All other Traα/β mutants had little or no effect on the extent of dsx RNA splicing, indicating that the C-terminal RS domain and RRM were essential for activity.

**Both RS domains of Tra2ß are active in stimulating RNA splicing**

In light of the dramatic differences in activity of Tra2β∆N and Tra2β∆C in modulating RNA processing of all the target RNAs tested (HIV-1 and dsx∆434), it was of interest to determine whether the differences might be attributable to differences in the relative activities of the RS domains. To address this question, either the N-terminal or C-terminal RS domain of Tra2β was fused to the C-terminus of the MS2 coat protein. Constructs were transfected into cells and expression confirmed by western blot (Fig. F in File S1) and immunofluorescence localization (Fig. F in File S1). As shown, both fusion proteins were expressed and accumulated in the nucleus although the fusion with the C-terminal RS domain was expressed at a slightly lower level. Subsequent functional tests upon cotransfection with constructs expressing dsx RNA with (pAdMLdsx MS2) or without (pAdMLdsx∆434) MS2 binding sites (Fig. F in File S1) revealed that enhancement of dsx RNA splicing was dependent upon the presence of MS2 binding sites in the RNA and, in agreement with earlier findings [57], enhancement of splicing was observed upon expression of either RS domain fused to the MS2 coat protein. Therefore, both RS domains are functional in this context.

**Supplementary Figures**

**Figure A. Pattern of HIV-1 RNA splicing.**

Shown at the top is the organization of the HIV-1 proviral genome indicating the position of the multiple 5’ splice donor sites (SD1 to SD4) and 3’ splice acceptor sites (SA1 to SA7) used in splicing of pre-mRNA. In the middle is an illustration of the alternatively spliced RNAs generated by processing of the HIV-1 genomic RNA. Indicated are the common (open boxes) and alternative exons (closed boxes) used in the generation of the SS (4 kb) and MS (1.8 kb) viral RNAs. At the bottom is a list of the nomenclature used to refer to the exon composition of the individual RNAs generated for both the SS and MS classes of HIV-1 RNAs.

**Figure B. Effect of Tra2α/β on Splice Site Usage in HIV-1 SS RNA.**

293T cells were transfected with pHxb2 R-/RI- with the indicated myc-expression vectors. RNA was extracted, cDNA generated and amplification of HIV-1 SS RNA performed as outlined in “Materials & Methods”. Shown in (A) is a representative gel of the results obtained. The products generated are identified on the left. (B) Summary of the relative abundance of splice products averaged over multiple assays. See Fig. S1 for a description of the products generated.

**Figure C. Mutation of the Tra2β RRM Inhibits its Modulation of HIV-1 MS RNA Splicing.**

293T cells were transfected with pHxb2 R-/RI- with the indicated myc-expression vectors. RNA was extracted, cDNA generated and amplification of HIV-1 MS RNA performed as outlined in “Materials & Methods”. Shown is a representative gel of the results obtained. The products generated are identified on the left. See Fig. S1 for a description of the products generated.

**Figure D. Effect of GFP-SR protein fusions on HIV-1 Gag Expression**

293 cells were transfected with pHxb2 R-/RI- with the indicated GFP-tagged expression vectors. 48 h post-transfection, cells were harvested and protein extracts fractionated on SDS-PAGE gels and blots probed with (A) anti-HIV-1 Gag antibody, (B) antibody to GAPDH or (C) GFP signal directly imaged.

**Figure E**. **Effect of** **Tra2α and Tra2β Overexpression on dsx RNA Splicing**

1. Schematic of the pAdML dsxΔ434 and pAdML dsx (GAA)_6_ expression constructs used to assess the effect of hTra2α/ß or mutants thereof. DsxF and dsxR are the primers used for the RT-PCR analysis and the positions are as indicated. AdML=adenovirus major late promoter.
2. 293T cells were transfected with 1 µg of either pAdML dsxΔ434 or pAdML dsx(GAA)_6_ and 4 µg of vectors expressing hTra2α, hTra2β or mutants thereof. Forty-eight hours post-transfection, cells were harvested, total RNA extracted, and RT-PCR performed. Amplicons were subsequently fractionated on native PAGE gels and amplicons corresponding to unspliced (US) and spliced (S) dsx RNA detected by exposure to phosphor screens. A representative example of the results of such analysis is shown as well as a summary of the results from at least three independent trials. Signals were quantitated from phoshor scans and ratio of unspliced to spliced RNA (US/S) calculated. To facilitate comparison between multiple assays, all values are expressed relative to the US/S ratio seen upon co-transfection with the control plasmid CMVmyc (set to 1).

## Figure F. Both RS Domains of Tra2β can enhance RNA splicing

A) Schematic of constructs. To evaluate the independent activities of the two RS domains of Tra2β, the regions encoding the N terminal (βRS-N) and C-terminal (βRS-C) RS domains were cloned 3’ of the NLS HAMS2 gene, encoding HA tagged MS2 RNA binding protein. Also shown is the structure of pAdML dsxMS2, indicating the position of insertion of the multiple MS2 binding sites.

B) Western blot and C) immunofluorescent localization of fusion proteins. 293T cells were transfected with CMV NLS HAMS2, CMV NLS HAMS2 ßRS-N or CMV NLS HAMS2 ßRS-C Western blots were performed to determine the level of protein expression and immunofluorescence to assess impact on protein localization. Fusion proteins were detected by anti-HA antibody.

D) Effect of RS Fusion Proteins on dsx RNA Splicing. pAdML dsx∆434 or pAdML dsx MS2 were co-transfected with CMV NLS HAMS2, CMV NLS HAMS2 βRS-N or CMV NLS HAMS2 βRS-C. RNA was harvested and extent of RNA splicing assessed by RT-PCR followed by fractionation on native polyacrylamide gels. Shown is a summary of results from multiple experiments (data expressed as unspliced to spliced (US/S) dsx RNA ratio). Asterisks denote samples that were significantly different from NLS HAMS2 (p<0.05).
